# Assessment of the Use of Food Supplements by Military Personnel: Study Protocol and Results

**DOI:** 10.3390/nu15081902

**Published:** 2023-04-14

**Authors:** Igor Pravst, Živa Lavriša, Hristo Hristov, Maša Hribar, Sanja Krušič, Katja Žmitek, Anita Kušar, Katja Zdešar Kotnik, Petra Golja, Anja Čibej Andlovec, Larisa Pograjc

**Affiliations:** 1 Nutrition Institute, Koprska ulica 98, SI-1000 Ljubljana, Slovenia; ziva.lavrisa@nutris.org (Ž.L.); hristo.hristov@nutris.org (H.H.); masa.hribar@nutris.org (M.H.); sanja.krusic@nutris.org (S.K.); katja.zmitek@vist.si (K.Ž.); anita.kusar@nutris.org (A.K.); 2 VIST–Faculty of Applied Sciences, Gerbičeva Cesta 51A, SI-1000 Ljubljana, Slovenia; 3 Biotechnical Faculty, University of Ljubljana, Jamnikarjeva 101, SI-1000 Ljubljana, Slovenia; katja.zdesarkotnik@bf.uni-lj.si (K.Z.K.); petra.golja@bf.uni-lj.si (P.G.); 4 Ministry of Defence of the Republic of Slovenia, Vojkova Cesta 55, SI-1000 Ljubljana, Slovenia; anja.cibej.andlovec@mors.si (A.Č.A.); larisa.pograjc@mors.si (L.P.)

**Keywords:** food supplements, dietary supplements, functional foods, nutrition, nutrient intakes, military, armed forces, sport nutrition

## Abstract

Due to their specific mode of operation, military personnel are challenged physically as well as mentally. In most countries, the use of food supplements by military personnel is not regulated, and a high prevalence of supplementation is expected. However, data on this are scarce or very limited, without insights into the importance of supplementation for the intake of bioactive substances. Our goal was, therefore, to develop a study protocol to enable an assessment of the prevalence of using food supplements and an estimate of the contribution of supplementation practices to the dietary intake of specific nutrients and other compounds. The protocol was tested in a study of Slovene Armed Forces (SAF) personnel. Data were collected using an anonymous questionnaire in a sample of 470 participants from different military units—about half from the barracks located across the country, and the other half returning from military operations abroad. To provide meaningful results, we recorded the use of food supplements and functional foods available in single-sized portions (i.e., energy drinks, protein bars, etc.). Altogether, 68% of the participants reported supplementation, most commonly with vitamin, mineral, and protein supplements. Military rank, participation status in military operations, and physical activity were the main determinants of the specific supplements used. Surprisingly, a lower prevalence of overall and protein supplementation was observed in subjects returning from military operations abroad (62 vs. 74%) than in personnel stationed in barracks across Slovenia; however, the frequency of the use of energy drinks and caffeine supplements was higher in this population (25 vs. 11%). The study design allowed for estimations of the daily intake of supplemented bioactive compounds. We describe the challenges and approaches used in the study to support similar studies in the future and within other populations.

## 1. Introduction

Dietary supplementation with vitamins, minerals, and other bioactive substances is widespread in various population groups [1,2,3,4,5,6,7,8].

While people typically decide to supplement their diets to improve inadequate nutrition and for various health-promoting reasons [9,10,11], specific populations may also look for effects on their physical and mental performance [12,13,14].

It is, therefore, not surprising that the use of food supplements can be more common in both athletes [15,16,17] and military personnel than in the general population [18].

Military personnel are a very diverse population group. While some employees mainly perform sedentary work, others are often exposed to prolonged and intense physical activity similar to that of professional athletes. Military personnel, especially those who are deployed in military operations abroad, often work in hostile environments under extreme conditions. This also affects their daily nutrient and energy requirements. Dietary recommendations for the Slovenian Armed Forces (SAF) [19] were established in 2017. A dietary survey of SAF members [20] revealed that, while the nutritional composition of meals was in compliance with these recommendations, some soldiers did not consume enough food to meet their dietary needs [21]. Dietary supplementation is not defined in the nutritional recommendations of the SAF [7,8,19] and has not yet been investigated.

According to European Union (EU) regulations [22,23], food supplements are concentrated sources of specific bioactive substances (substances with a nutritional or physiological effect)that are intended to complement daily nutrition and are available in small dosage forms, such as pills, powders, capsules, and so on. However, people can supplement their diets with products which are, regulatorily, not considered food supplements. A particularly important source of dietary bioactive substances is enriched functional foods. For example, protein bars can present a notable source of proteins [24], and energy drinks can contribute to the intake of caffeine and some other substances [25]. It, therefore, makes sense for the evaluation of dietary supplementation practices not to be strictly limited to the use of food supplements but to also account for other products used by consumers for similar purposes.

In a few countries, dietary supplementation practices in specific military personnel populations have been investigated to at least some extent. For example, supplementation is notably more prevalent in US military personnel than in the general population [13,18]. Caldwell et al. [26] presented a survey instrument to investigate the use of food supplements and related products in high-use populations, but this tool was adapted to the availability of specific products in the USA. Similar studies have been carried out in population groups from Australia [27], Germany [28], and the UK [14]. However, the overall data regarding dietary supplementation practices in military personnel are quite limited and do not provide insights into how many nutrients and other substances are supplemented. It should be noted that such data are not available for Slovenia.

Considering these challenges, our goal was to develop a study protocol to enable the assessment of the prevalence of dietary supplementation and an estimate of the contribution of supplementation practices to the dietary intake of specific nutrients and other bioactive compounds. The protocol was then used in a study of SAF personnel, using participants from barracks located across the country and from those returning from military operations abroad. The latter group was included because operations abroad are carried out in jurisdictions with different availability and regulatory statuses of supplements, and higher use of supplements was expected due to the specific working conditions. The assessment of the supplementation practices included the use of food supplements and enriched functional foods that are available in single portion sizes (such as energy drinks and protein bars), but not the use of unauthorized or illegal substances. Therefore, this paper covers food supplements and similar products (FSSPs) that are enriched with nutrients or other bioactive substances and are marketed for use in single portion sizes.

## 2. Materials and Methods

### 2.1. Survey Instrument

Building on the approach developed by Lieberman et al. for the cross-sectional measurement of the prevalence of dietary supplementation [13], we developed a survey instrument that enabled the collection of more detailed information on the composition and level of consumption of the FSSPs. The tool was designed as an anonymous questionnaire for single administration and is presented in the Supplementary Materials (see Survey Tool ST1). The tool was organized into three sections:

The demographic and general data section (1) included sex, year of birth, body mass, height, rank/title, education level, and the Army Physical Fitness Test (APFT 1-5) [29]. All data were self-reported without measurements.

The physical activity assessment section (2) included the International Physical Activity Questionnaire (IPAQ) [30], which was adapted into a graphical form. In short, we asked the participants how many hours and minutes they were active—vigorously, moderately or low-intensively—during each day in the past week. Vigorous-intensity activity was described as an activity where the heart rate greatly increased, breathing was heavier, and sweating was more intense, such as running, fast swimming, fast cycling, and other similar activities. Moderate physical activity included any form of activity where heart rate and breathing moderately increased, such as cycling and jogging. Low-intensity physical activity included walking and other activities where heart rate and breathing did not increase.

- The section on the use of FSSPs (3) was divided into three subsections:
- (3A) The subjects were first introduced to a definition of an FSSP. Following the protocol of Zdešar Kotnik et al. [31,32], the subjects were then asked whether they had consumed any such products within the last 12 months. We also asked them where they purchased the FSSPs (multiple answers were possible). Only the users of FSSPs were asked to continue to the next subsection.
- (3B) The table collection of all consumed FSSPs included: the product and manufacturer’s name, the product form (capsule, powder; tablet; syrup; gel; bar; fizzy tablet; drops; spray; plastic bottle/can; other), daily dose (e.g., 2 capsules), a URL link to the product details, and the availability of a product photo (yes/no).
- (3C) Details were collected for each reported FSSP. The subjects were asked to provide the frequency of the consumption of a specific product during each season (daily, 4–6 times per week, 1–3 times per week, 1–3 times per month, less frequently, or never), and their use of the product in specific situations (during a period which required greater physical capability, during a military operation abroad, during the assessment of movement skills, and during illness). The subjects were further asked to identify the reasons for using specific products (multiple answers were possible), who recommended they use the products, and their self-reported observations of any positive or negative effects of the use of the FSSPs.

### 2.2. Administration of Survey Tool

Prior to their enrolment in the research, the SAF informed potential subjects of the option to voluntarily participate. Details of the study were provided, and the subjects were informed about its objectives and the fact that they would need to provide details of their use of food supplements and similar products during the last 12 months. They were advised to take photographs of all the products that they were using to support the product identification process.

The study was conducted under the supervision of a researcher, not affiliated with the SAF, using a paper version of the survey tool. This meant that, prior to the start of data collection, the subjects were informed, in detail, about the scope and definition of FSSPs to make sure that all the relevant information was collected. Data collection was only carried out for subjects who signed a voluntary agreement form for their participation in the study. The researcher was available for support and consultation during the whole period of data collection, which was carried out either with individual subjects or with smaller groups of subjects in classrooms. In order to enable the correct identification of all the used FSSPs, together with their bioactive ingredients, the researcher was particularly careful to support the participants in completing section 3B, ensuring that as many details as possible were provided. While some subjects had photographs or labels of the products they used, mostly this was not the case; therefore, the subjects were asked to provide details of the product, preferably including URL links. This turned out to be very valuable, because the participants were able to find most products using their smartphones. The time required for the completion of the survey depended on the number of different products reported; most of the subjects completed the survey within 15 min. A few subjects who reported the use of several FSSPs needed more time. It should be noted that, due to the approach used (paper form), the survey time was not recorded in detail at the individual level.

### 2.3. Study Sample and Enrolment Details

The study was conducted with members of the SAF from January to December 2018. About half of the study sample originated from barracks across Slovenia, while the other half were personnel who had returned from military operations abroad. The subjects from the barracks were invited randomly, with no consideration of demographic parameters at the invitation stage. Data collection with these subjects was carried out in the barracks. Those returning from missions abroad were invited without randomization, using a first come, first served criteria. Data collection with this population was carried out in a military healthcare centre in addition to regular health checks. A total of 470 SAF personnel participated in the study, but 22 were excluded due to incomplete data. The final study sample, therefore, consisted of 448 participants.

### 2.4. Data Extraction

All completed questionnaires were subjected to data extraction into a digital form. For practical reasons, we used three inter-linked spreadsheets:

(A) A spreadsheet was developed to house data about the study subjects. This spreadsheet included all the data from sections 1–2 of the survey tool. Each subject was inserted in a new line with a unique subject identification number (SID).
(B) A spreadsheet was developed for data on the composition of all the reported food supplements and similar products. Different participants may have reported the use of the same FSSP, but each product was inserted into this spreadsheet only once. Each product was inserted in a new line with a unique food supplements identification number (FSID). These data originated from section 3A of the survey tool and were supplemented with detailed product compositions with the use of product labelling pictures (obtained from descriptions on the provided URL links) and from the national branded foods composition database, CLAS (Composition and Labelling Information System) [33]. Each FSSP was manually categorized. In the absence of an internationally harmonized categorization system for food supplements, a previously used categorization [13] was adapted and modified to include products similar to food supplements (vitamin–mineral supplements, vitamin supplements, mineral supplements, protein supplements, fatty acid supplements, energy drinks and caffeine supplements, sports drinks and similar creatine, herbal supplements, and other supplements). It should be noted that the food supplements were not subjected to laboratory analyses, and thus, the exact composition of the FSSP could not be verified. Altogether, 467 products were recorded, but 5 were designated as out of scope because they did not meet our definition of an FSSP (these included peanut butter, soya nuts, oatmeal, and two brands of baking powder). For the remaining 462 products, we were able to obtain detailed labelled composition data.
(C) A spreadsheet was developed for data on the use of food supplements and similar products. This spreadsheet presented a link between the specific subjects (SID) and the supplements used (FSID). Each reported use of a food supplement or similar product was recorded in a separate line. For example, if subject ID1 reported using two products, two lines were inserted—each specifying details of the FSSP, including daily dosage and seasonal frequency, the origin of the recommendation, and self-reported positive/negative effects of the FSSP.

### 2.5. Data Analysis

Self-reported body mass and height were used to calculate the body mass index (BMI) by dividing their body mass by the square of their height in metres. The subjects were grouped using the BMI cut-off points of <18.5, <25.0, and ≥25.0 kg/m^2^ for underweight, normal weight, and overweight, respectively [34]. Physical activity data were used for the calculation of daily energy expenditure for physical activity (DEEPA) [35]. DEEPA is defined as kcal used per kg of body mass per day and is calculated as DEEPA = [(hours of VPA × 6 MET) + (hours of MPA × 3 MET)]/7 days, where VPA is vigorous-intensity physical activity, MPA is moderate-intensity physical activity, and MET is standard metabolic equivalent, a unit used to estimate the amount of oxygen used by the body during physical activity (kcal·kg^−1^·day^−1^). The MET for VPA was considered to be 6 and, for MPA, the value was 3 [35]. Based on this, the subjects were assigned to one of the three categories. As proposed by Wong and Leatherdale [36], cut-off values were set using a standard deviation approach: subjects with a DEEPA more than one standard deviation (SD) below the average (in our study, DEEPA < 1.8 kcal·kg^−1^·day^−1^) were designated as (1) low physical activity, those with DEEPA higher than 1 SD above the average (in our study, DEEPA > 13.3 kcal·kg^−1^·day^−1^) were designated as (3) high physical activity, and the rest were designated as (2) moderate physical activity.

Descriptive characteristics (prevalence with 95% confidence intervals (CIs)) are presented for the overall use of supplements (any FSSPs) and for specific categories of FSSPs. Descriptive data were calculated as prevalences (%) and were further analyzed using relevant statistical methods. Logistic regression analysis was used to determine odds ratios (ORs) compared with the baseline level; the results are reported with 95% confidence intervals (CIs). ORs were calculated for the different levels of a variety of variables: age at three levels (18–29, 30–39, and 40 years and above); sex (male, female); military operation abroad (yes, no); education at three levels—lower secondary school (primary school or lower and lower secondary school or secondary specialized school), secondary school (four-year secondary school or secondary school), and higher education (short-cycle college, college or first cycle Bologna degree programme, university degree or second cycle Bologna degree programme, Master’s degree, or doctorate); and rank at three levels—private, non-commissioned officer, and higher ranks and others (warrant officer, commissioned officer, senior officer, general, junior military specialist, senior military specialist, other). Three levels were formed for the APFT score (3 or less, 4, and 5); body mass index (BMI; <25, 25–30, and above 30 kg/m^2^); and DEEPA score (low, moderate; high physical activity). Differences between levels were assessed using Pearson’s chi-squared test for equality of column proportions. Continuity correction was carried out using the Holm procedure [37]. Differences were considered significant at *p* < 0.05.

The intake of nutrients and other bioactive substances in FSSPs was estimated per day. It should be noted that estimated intake reflects only the use of FSSPs and does not account for regular dietary intake. Estimates were made for using all the reported products, and without corrections for seasonal differences. The intake of a substance with a particular FSSP was, thus, calculated as the mathematical product of the substance contained in a product’s dosage and the reported number of consumed dosages per single use, with the consideration of consumption frequency. The total intake of a particular substance for a subject was then calculated as the sum of the intake rates of this substance, with consideration for all reported FSSPs. The results are presented in histograms providing insights into the distribution of the intake of specific substances for those subjects in the study population who supplemented their diet with a given substance.

## 3. Results and Discussion

Of 448 subjects, the majority (89%) were male. The age of the participants was 21–60 years. Altogether, 54% of participants were privates and 29% were non-commissioned officers, while the remainder (17%) were those with higher military ranks and others. The majority of the participants (64%) had secondary school education, and about a quarter (26%) obtained higher education. More than half of the subjects (57%) reported the highest score on the Army Physical Fitness Test. Using the DEEPA scores, 12% of the subjects were assigned to the low physical activity group, 75% were assigned to the moderate group, and 13% were assigned to the high physical activity group. 

The prevalence of use of different types of FSSPs according to different socio-demographic, anthropometric, and physical activity characteristics and the place of military service is presented in Table 1. Altogether, 68% of the participants (*n* = 304) (67% of men; 71% of women) reported using FSSPs. Similar results were observed in the UK and the USA, where 69% of military personnel reported the use of supplements [18,38]. An even higher prevalence of dietary supplementation was reported for elite soldiers from the USA (87%) [39], probably because this group of military personnel is often involved in vigorous physical activity and performs duties in extreme conditions. Our data were not analyzed for different military units, but we conducted sub-group analyses with consideration for operations abroad and self-reported physical activity. A somewhat higher prevalence of the general use of FSSPs was observed in subjects with high physical activity (76%); surprisingly, the lowest prevalence was observed in subjects returning from military operations abroad (62%). However, the prevalence of using FSSPs in this group was comparable to British Army soldiers in training [14]. In our study, the participants who used FSSPs more frequently were privates (72%). In fact, military rank was one of the few variables where we observed statistically significant group differences (*p* = 0.003). Non-commissioned officers were, in general, significantly less likely to use FSSPs than privates (odds ratio (OR) = 0.38, *p* = 0.001). In general, typical users of FSSPs were those younger than 40 years with higher education, high physical activity, and a higher APFT score. These findings are in line with literature reports [18,38], where a higher prevalence of supplementation was particularly notable among more physically active participants and in those with higher education.

### 3.1. Use of Different Types of Food Supplements and Similar Products

**Figure 1:**
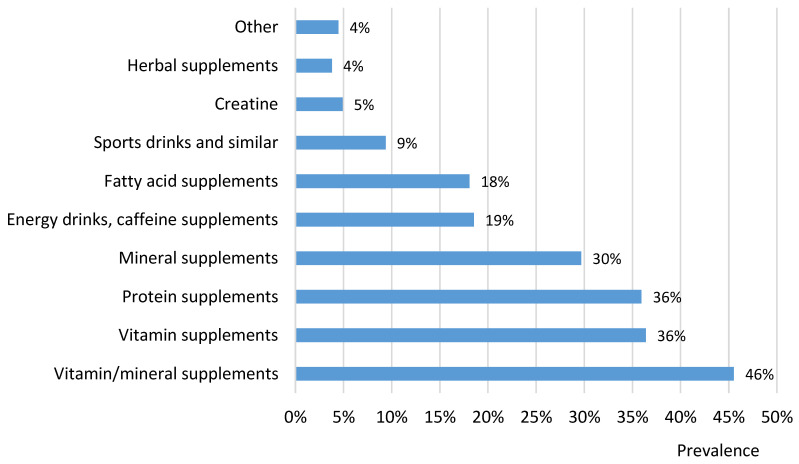
Prevalence of use of different types of food supplements and similar products among participants recruited from the Slovenian Armed Forces during a 12-month period (*n* = 448).

To provide further insights, we assessed the usage of different types of FSSPs. The prevalence is shown in Figure 1, while further details are provided in Table 1 and Supplementary Table S1. Self-reported reasons for using FSSPs are presented in Supplementary Table S2. Considering the 12-month observation period, the highest prevalence of using FSSPs was recorded for combinations of vitamins and minerals (46%), followed by vitamin and protein supplements (both 36%) and mineral supplements (30%) (Figure 1). These findings are very similar to the results of other studies on supplementation in military personnel [12,13,38,40], which have highlighted high prevalences of vitamin, mineral, and protein supplement use. According to a literature review [6], vitamins and minerals, whether taken in combination or individually, are the most frequently consumed dietary supplements by military personnel, and this was also the case in our population. Our results revealed that the SAF participants who served at home and those with a higher APFT score reported a significantly higher prevalence of the use of vitamin and mineral FSSPs (*p* < 0.05). The most frequently reported reasons for taking vitamin–mineral supplements were the improvement of immune system function (66%) and compensation for the inadequate intake of certain nutrients in a regular diet (60%) (Supplementary Table S2). This is in compliance with reasons stated in some studies of the general population, where vitamin–mineral supplements are used mostly to improve general health and fill nutrient gaps in diets [41,42]. The high prevalence of such products can be explained by convenience; vitamin–mineral supplements commonly contain multiple ingredients, and consumers can perceive these as a single solution for an unbalanced diet or for better general health [43].

Among vitamin supplements, we mostly observed the use of multivitamin supplements. Within single vitamin supplements, we observed supplementation with vitamin C or vitamin D. This may be partially explained by the expectations of users that such products are beneficial for the functioning of the immune system. In the EU, both vitamins C and D are associated with authorized immunity-related health claims, which can be used on food supplements (‘contributes to the normal function of the immune system’) [44]. There were no recent data available about the intake of these vitamins and deficiencies in members of the SAF, but it has been reported in the past that their intake of selected vitamins is adequate [20]. While we can assume that the dietary intake of vitamin C is sufficient [45], it should be mentioned that a very low dietary intake of vitamin D [46,47] and a high prevalence of vitamin D deficiency [48] have been determined among the general population. However, these results were publicly communicated [49] after the conduct of this study and, therefore, did not affect our results.

The study participants mostly stated that their use of mineral supplements was for body regeneration (75%). Magnesium supplements were the only single mineral FSSP worth mentioning. This was expected because magnesium is widely used among athletes and those with higher physical activity for reasons including its potential to enhance muscle recovery after exercise [50].

Another commonly used group of supplements in our study was protein supplements (36%, Figure 1). A typical example of such a product is protein powder (e.g., whey isolates). These products can be labelled and marketed with the authorized health claim ‘contributes to growth of muscle mass’ [44]. Requirement levels for protein intake may be higher in athletes and those who perform strength training due to extra protein required for muscle synthesis, although a review of studies showed that such a requirement increase is only modest and can, in some cases, even be unnecessary [51,52]. The nutritional recommendations for protein intake for SAF personnel are indeed higher than those for the general population, i.e., 1.2 g/kg of body mass daily for those with low physical activity levels and 1.8 g/kg of body mass daily for those engaging in more intensive physical activity [19], while a daily intake of 0.8 g/kg of body mass is recommended for the general population [53]. We observed a significantly higher prevalence of protein supplement use among non-commissioned officers (in comparison with privates and higher ranks). The study participants explained that they were using these products for recovery (79%) and to increase physical capability (strength and muscle mass gain) (68%). According to the literature [54], we expected to observe the use of creatine supplements. While the prevalence was not very high (5%), it should be noted that creatine was also contained in some other groups of FSSPs.

Energy drinks and caffeine-containing FSSPs were quite a prevalent product group (19%). The most commonly reported reasons for their use were the prevention of drowsiness (61%) and reducing fatigue (60%). We observed a significantly higher prevalence of energy drinks and caffeine-containing FSSP use in the subgroup of subjects returning from military operations abroad (25%), in those younger than 30 years (29%), in those with lower physical activity (37%), and in those with a lower education level (25%) (Table 1). Aside from energy drinks, 9% of all participants used sports drinks and similar products, such as isotonic drinks. Rehydration (66%) was most often mentioned as the reason for using such products.

Another quite commonly reported group of FSSPs was fatty acid supplements (18%). These were mostly products with omega-3 fatty acids, particularly fish oil and supplements containing docosahexaenoic acid (DHA) and/or eicosapentaenoic acid (EPA). Furthermore, 4% of participants reported using different herbal supplements.

In our survey, we also asked the participants where they bought FSSPs. The question was non-specific, without details for specific groups of supplements. Altogether, about half of the participants reported that they bought FSSPs in food stores (49%). This could be explained by the fact that our definition of FSSPs included energy drinks, sports drinks and some other products that are commonly available in food stories. Other important sources of FSSPs were online stores (32%) and specialized stores (29%). Only 19% reported buying FSSPs in pharmacies, where professional consultation is available. When buying dietary supplements online, one relies only on commercial information provided on websites, which sometimes includes very aggressive marketing techniques [55].

### 3.2. Food Supplements and Similar Products as Sources of Nutrients and Other Biologically Active Ingredients

**Figure 2:**
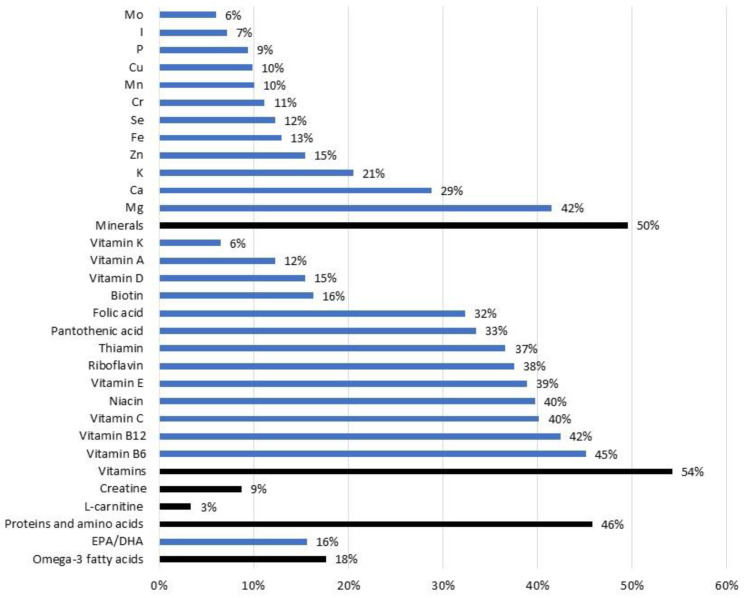
Prevalence of supplementation with specific nutrients and other biologically active ingredients for study participants recruited from the Slovenian Armed Forces during a 12-month period (*n* = 448). Note: the black lines indicate specific categories, and the blue lines indicate their subcategories.

**Figure 3:**
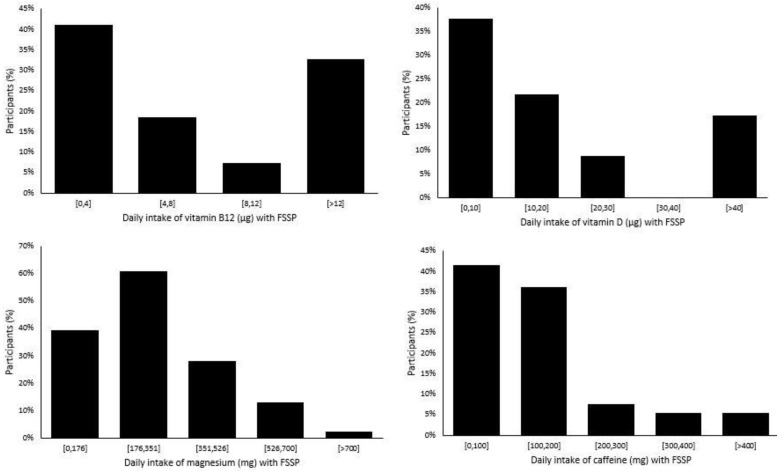
Daily intake of specific ingredients of food supplements and similar products (FSSPs) among study participants recruited from the Slovenian Armed Forces.

The study participants reported the use of a variety of different supplements, which can be sources of the same ingredients. For example, a specific vitamin can be present in vitamin/mineral supplements, vitamin supplements, sports drinks and so on. We, therefore, assessed the prevalence of supplementation with specific nutrients and other biologically active ingredients and estimated the dietary intakes of these constituents. In this paper, we focused on the key results (Figure 2 and Figure 3), while additional details are presented in Supplementary Table S3.

Altogether, 54% of the study population was taking vitamin-containing FSSPs. The most prevalent supplemented vitamins were B6 (45%) and B12 (42%) (Figure 2), which are both very common in multivitamin supplements. Vitamin B6 is very abundant in many foods, and deficiency is not likely. While the SAF recommendations for B6 intake are somewhat higher than those for the general population [19], it is not likely that this was the reason for the higher prevalence of supplementation with this vitamin in the study population. We should note that the prevalence of vitamin B12 deficiency is low in the Slovenian general population [56]. The most common causes of vitamin B12 deficiency are malabsorption, which is more common in older adults, or inadequate intake, particularly in those who exclude foods of animal origin from their diet. However, a vegan/vegetarian diet is not common among SAF members [20]. It should be mentioned that, in the European Union, supplements containing vitamin B12 can be marketed with a series of strong, authorized health claims referring to the nervous system (i.e., contributes to a reduction in tiredness and fatigue, psychological function, and the functioning of the nervous system) [44], making such products very interesting for consumers. Some food supplements contain quite high quantities of vitamin B12, and this can also be seen in the estimated intake levels in our study. The recommended daily intake of vitamin B12 for members of the SAF is 3.0 µg [19], while in our study, the median intake of vitamin B12 with FSSP only (thus, among users) was 5.0 µg. Nevertheless, the average intake was 35 ± 147 µg (SD), because more than one-third of those who were supplementing vitamin B12 were using more than 12 µg of vitamin B12 (Figure 3). Overall, 10 of our subjects supplemented ≥100 µg, and 4 used ≥1000 µg vitamin B12.

While a deficiency of the B vitamin group is very rare [56], the contrary is the case for vitamin D. About 40% of Slovenian adults are vitamin D deficient during winter time [48], so supplementation would be beneficial among members of the SAF. Surprisingly, only 15% of our study population supplemented their diet with vitamin D. The recommended daily intake for members of the SAF is 20 µg [19]. While the average intake of FSSPs in our study was 21.5 ± 29.8 µg, the median intake was 12.5 µg, and over 40% of users were supplementing less than 10 µg of vitamin D. This is due to the fact that, while the typical dosage of single vitamin D supplements is about 20 µg [49], many multi-vitamin products contain much smaller quantities of vitamin D because the EU food labelling reference for 100% RDA (recommended daily allowance) is set at only 5 µg [57].

The overall prevalence of supplementation with minerals in our population group was 50%, most commonly with magnesium (42%), followed by calcium (29%). The recommended daily intake of magnesium for members of the SAF is 350–400 mg [19], and previous studies have shown adequate magnesium intake from military meals in Slovenia [20]. We determined that, in those subjects who supplemented their diet with magnesium, the average daily supplementation dose was 293 ± 402 mg, with a median dose of 200 mg. In Europe, the tolerable upper intake level (UL) for the daily intake of magnesium from supplements (in addition to regular dietary intake) has been set at 250 mg [58]. While the typical intake of magnesium in our study was lower, our study highlighted that about 18% of the study population was at risk of exceeding the UL.

We also estimated the intake levels of caffeine with FSSP. Altogether, 21% of the study participants were using such products, mainly in the form of energy drinks. Well-established evidence exists regarding the effects of caffeine supplementation on cognitive function, attention and vigilance, complex reaction time, and so on [59]; such supplementation can, therefore, be expected among military personnel [60,61]. Energy drinks can contain high levels of caffeine, sometimes even more than 500 mg of caffeine per bottle or consumption unit [62]. Excessive consumption can, therefore, pose a health risk because of the high caffeine level and because of other constituents. Possible undesired effects include anxiety, arrhythmia, and gastrointestinal issues [63]. Our study also investigated undesirable side effects of using FSSPs, and energy drinks were practically the only group where such effects were reported. Reported effects included gastrointestinal upset (9%), headache (2%), cardiovascular problems (3%), and worse feelings in general (5%). The mean caffeine supplementation in our study was 198 ± 282 mg, with a median of 170 mg. According to a European Food Safety Agency (EFSA) report, single doses of caffeine of up to 200 mg and daily doses of up to 400 mg do not raise safety concerns [64]. Only five of our study participants exceeded this limit (Figure 3), but it should be noted that we did not collect data on the consumption of other food and drink that may contain caffeine and could represent an important dietary source of caffeine (e.g., coffee).

It is worth mentioning some other active ingredients which were present in the reported FSSPs. About 18% of our study sample reported supplementing their diets with omega-3 fatty acids (16% with EPA or/and DHA). The usage of creatine was quite prevalent (9%). We also observed supplementation with various botanical ingredients (altogether, 15%): lutein, zeaxanthin and/or astaxanthin (4%), lycopene (4%), flavonoids and/or polyphenols (3%), L-carnitine (3%), and coenzyme Q10 (2%).

### 3.3. Study Strengths and Limitations

The strength of this study is in its quite large study sample and in the fact that data collection was linked to the use of specific food supplements and similar products. Previous studies have mostly focused on the assessment of the prevalence of use of selected groups of food supplements or selected predefined products, while our approach was wider, providing additional insights into supplementation with specific substances. A limitation of such an approach is that all the reported products need to be linked to detailed product composition. We overcame this challenge with a careful data collection approach, which included on-site support for the study participants and the identification of reported FSSPs on the internet during surveying. While this approach was possible with the in-person surveying method we used, this might not be feasible in online surveys. Another limitation is that our study was only focused on supplementation, and we did not estimate the intake of nutrients and other substances with regular foods. While food consumption data would have enabled us to estimate total dietary intake levels, this would have made the study unfeasible within the available budget and would pose a major burden on the study participants. We need to mention that our intake estimations were carried out with the end-limit scenario of using multiple products without corrections for seasonal differences and may, therefore, have resulted in somewhat overestimated values in specific cases. For example, some subjects reported the use of different brands of similar FSSPs. While this did not affect the prevalence estimates for the use of specific types of FSSPs, estimates of the daily intake of the constituents of such products have, consequently, been affected. Furthermore, we should note that the survey question about where the participants purchased FSSPs was quite general since it was not related to our primary goals. More meaningful results on this topic would be available if this question were linked to specific reported products.

## 4. Conclusions

Our study demonstrated a high prevalence of using food supplements and similar products among members of the Slovenian Armed Forces. The most commonly used products were vitamins, minerals, and proteins, followed by caffeine supplements/energy drinks and fatty acid supplements. With the exception of caffeine supplements/energy drinks, a lower prevalence of use was observed in subjects returning from military missions abroad. We showed that the study methodology used enabled the estimation of the intake levels of constituents of food supplements. This is particularly valuable for the assessment of benefits and risks related to supplementation practices. It should be mentioned that supplementation practices are very closely related to personal knowledge in the area of nutrition and health [49,65]. Individuals lacking nutrition knowledge can be more easily misled into the (unnecessary) use of food supplements, which can result in undesirable effects on general health and the waste of money [66,67]. Further studies in this area should, therefore, also focus on the identification of knowledge gaps related to dietary supplementation. Military personnel are a specific population group, facing demanding physical and mental challenges during military training and operations. In such circumstances, dietary supplementation may be beneficial; however, considering that only a few supplementation approaches can be scientifically supported, education tools should be developed to support better-informed usage of such products.

## Figures and Tables

**Table 1:** Prevalence of use of food supplements and similar products (FSSPs) and odds ratios (95% confidence interval) for study participants recruited from the Slovenian Armed Forces (*n* = 448).

Variable		Participants		Use of: Any FSSP			Vitamin and Mineral FSSPs			Protein FSSPs			Energy Drinks and Caffeine FSSPs		
		*n*	*n* (%)	*n*	*n* (%)	Odds Ratio	*n*	*n* (%)	Odds Ratio	*n*	*n* (%)	Odds Ratio	*n*	*n* (%)	Odds Ratio
Overall		448		304	68		204	46		163	36		83	19	
Age	18–29	85	19	65	76	1	42	39	1	36	35	1	20	29 ^b^	1
	30–39	196	44	149	76	1.30 (0.71–2.36)	88	54	0.95 (0.55–1.65)	77	32	1.02 (0.59–1.78)	42	21 ^b^	0.64 (0.32–1.26)
	≥40	167	37	90	54	1.69 (0.84–3.39)	74	39	1.12 (0.60–2.13)	50	41	0.75 (0.39–1.45)	21	10 ^a^	0.39 (0.17–0.93)
Sex	Male	399	89	269	67	1	184	45	1	146	37	1	74	19	1
	Female	49	11	35	71	0.85 (0.43–1.66)	20	53	0.77 (0.40–1.47)	17	31	0.88 (0.45–1.73)	9	12	0.87 (0.37–2.03)
Education	Lower secondary school	44	10	29	66	1	20	30	1	15	39	1	16	25 ^b^	1
	Secondary school	287	64	189	66	1.02 (0.49–2.16)	133	44	1.10 (0.56–2.15)	110	37	1.39 (0.69–2.82)	49	17 ^a^	0.40 (0.18–0.84)
	Higher education	117	26	86	74	0.92 (0.37–2.25)	51	55	1.11 (0.49–2.53)	38	33	0.93 (0.39–2.25)	18	20 ^b^	0.51 (0.19–1.36)
Rank	Private	244	54	176	72 ^b^	1	121	44	1	100	32 ^b^	1	53	21	1
	Non-commissioned officer	128	29	74	58 ^a^	0.38 (0.22–0.67)	51	43	0.63 (0.37–1.06)	35	43 ^a^	0.57 (0.33–0.99)	21	13	1.12 (0.56–2.29)
	Higher ranks/Others	76	17	54	71 ^b^	0.49 (0.22–1.07)	32	54	0.71 (0.34–1.46)	28	37 ^b^	1.25 (0.58–2.69)	9	21	0.62 (0.22–1.76)
Operation abroad	Yes	229	51	141	62	1	95	36 ^b^	1	84	29	1	42	25 ^a^	1
	No	219	49	163	74	1.06 (0.70–1.62)	109	55 ^a^	1.47 (0.99–2.18)	79	43	0.95 (0.63–1.43)	41	11 ^b^	1.16 (0.69–1.95)
APFT score	3 or less	89	20	49	55	1	36	39 ^b^	1	37	39	1	18	11	1
	4	102	23	60	59	0.87 (0.46–1.64)	44	40 ^ab^	1.36 (0.74–2.50)	31	32	0.70 (0.37–1.32)	22	18	1.14 (0.53–2.48)
	5	257	57	159	62	1.15 (0.65–2.03)	124	50 ^a^	1.75 (1.02–2.98)	95	37	1.02 (0.59–1.75)	43	21	0.87 (0.43–1.75)
BMI	<25	127	28	91	72	1	44	50	1	32	36	1	20	17	1
	25–30	260	58	174	67	1.36 (0.78–2.22)	129	43	1.01 (0.61–1.68)	103	35	1.09 (0.64–1.85)	55	19	0.91 (0.48–1.71)
	>30	61	14	39	64	1.57 (0.74–3.36)	31	48	1.12 (0.49–2.53)	28	41	1.28 (0.62–2.63)	8	20	0.44 (0.16–1.21)
Physical activity	Low	54	12	37	69	1	19	35	1	25	54	1	20	37 ^b^	1
	Moderate	335	75	222	66	0.88 (0.47–1.67)	155	46	1.44 (0.78–2.66)	115	34	0.58 (0.32–1.06)	58	17 ^a^	0.29 (0.15–0.57)
	High	59	13	45	76	1.33 (0.56–3.18)	30	51	1.51 (0.68–3.33)	23	39	0.66 (0.30–1.46)	5	8 ^a^	0.13 (0.04–0.41)

Notes: APFT, Army Physical Fitness Test; physical activity group was assigned using DEEPA (daily energy expenditure for physical activity) scores; group differences were assessed using Pearson’s chi-squared test for the equality of column proportions; *p*-values were adjusted using the Holm correction method; different superscript letters indicate significant differences at *p* < 0.05.

## Data Availability

The data presented in this study are available upon request from the corresponding author. Sharing the data is subject to the approval of the study funder (the Ministry of Defence of the Republic of Slovenia).

## Supplementary Materials

The following supporting information can be downloaded at: https://www.mdpi.com/article/10.3390/nu15081902/s1, Supplementary Survey Tool ST1, Supplementary Table S1: Number and frequency of study participants who used supplements based on demographic and anthropometric data (n = 448), Supplementary Table S2: Self-reported reason for using food supplements and similar products (FSSP) for study participants recruited from the Slovenian Armed Forces, Supplementary Table S3. Prevalence of use of food supplements and similar products (FSSP) and odds ratios (95% confidence interval) for study participants recruited from the Slovenian Armed (12-month period).
