# Supplementary material for: Assessment of the Use of Food Supplements by Military Personnel: Study Protocol and Results
Source: Nutrients. 2023 Apr 14;15(8):1902. doi: 10.3390/nu15081902 (PMC10145590; doi:10.3390/nu15081902)
Supplement: Supplementary file 1 [file nutrients-15-01902-s001.zip › nutrients-2324109-supplementary.pdf]

Supplementary Survey Tool ST1

1 DEMOGRAPHIC AND GENERAL DATA

1.1 Sex:

1.1.1 Male

1.1.2 Female

1.2 Year of birth: ________

1.3 Body mass: ________ kg

1.4 Height: ________ cm

1.5 Rank or title:

1.5.1 Private
1.5.2 Non-commissioned officer
1.5.3 Warrant officer
1.5.4 Commissioned officer
1.5.5 Senior officer
1.5.6 General
1.5.7 Junior Military Specialist
1.5.8 Senior Military Specialist
1.5.9 Other: _______________________

1.6 What is the highest degree of education you have completed?

1.6.1 Primary school or lower
1.6.2 Lower secondary school or secondary specialized school
1.6.3 Four-year secondary school
1.6.4 Secondary school (general upper secondary school, secondary technical school, secondary school of economics, etc.)
1.6.5 Short-cycle college, college or First Cycle Bologna Degree Programme
1.6.6 University degree or Second Cycle Bologna Degree Programme
1.6.7 Master's Degree or Doctorate

1.7 Your last Army Physical Fitness Test (APFT) assessment mark? (Please, circle):

1.7.1 1

1.7.2 2

1.7.3 3

1.7.4 4

1.7.5 5

1.7.6 I have not had my APFT assessed.

2 PHYSICAL ACTIVITY ASSESSMENT

NOTE: If you have been absent in the last 7 days due to sick leave or paid leave, please consider the 7 days prior to the leave when completing the questionnaire.

2.1 Demanding physical activity

Please mark the hours and minutes of **VIGOROUS physical activity** you carried out **in the last 7 days**.

		Hours					Minutes			
2.1.1	Monday	0	1	2	3	4+	0	15	30	45
2.1.2	Tuesday	0	1	2	3	4+	0	15	30	45
2.1.3	Wednesday	0	1	2	3	4+	0	15	30	45
2.1.4	Thursday	0	1	2	3	4+	0	15	30	45
2.1.5	Friday	0	1	2	3	4+	0	15	30	45
2.1.6	Saturday	0	1	2	3	4+	0	15	30	45
2.1.7	Sunday	0	1	2	3	4+	0	15	30	45

Please include demanding physical activity during working hours, leisure time and sports training. Consider running, fast cycling, fast swimming, team sports, fast dancing and other activities which require major effort**; your heart rate and breathing increase severely and you begin to sweat.**

2.2 Moderate physical activity

Please mark the hours and minutes of **MODERATE physical activity** you carried out **in the last 7 days**.

		Hours					Minutes			
2.2.1	Monday	0	1	2	3	4+	0	15	30	45
2.2.2	Tuesday	0	1	2	3	4+	0	15	30	45
2.2.3	Wednesday	0	1	2	3	4+	0	15	30	45
2.2.4	Thursday	0	1	2	3	4+	0	15	30	45
2.2.5	Friday	0	1	2	3	4+	0	15	30	45
2.2.6	Saturday	0	1	2	3	4+	0	15	30	45
2.2.7	Sunday	0	1	2	3	4+	0	15	30	45

Please include commuting to and from work, activity during working hours and leisure time.
Consider fast walking, slow cycling, slow swimming and other activities which require moderate effort**; your heart rate and breathing are slightly increased.**

2.3 Walking

Please mark the hours and minutes of **WALKING** you carried out **in the last 7 days**. Consider walking that has been carried out for **at least 15 minutes at the same time**.

		Hours					Minutes			
2.3.1	Monday	0	1	2	3	4+	0	15	30	45
2.3.2	Tuesday	0	1	2	3	4+	0	15	30	45
2.3.3	Wednesday	0	1	2	3	4+	0	15	30	45
2.3.4	Thursday	0	1	2	3	4+	0	15	30	45
2.3.5	Friday	0	1	2	3	4+	0	15	30	45
2.3.6	Saturday	0	1	2	3	4+	0	15	30	45
2.3.7	Sunday	0	1	2	3	4+	0	15	30	45

Please include commuting to and from work, activity during working hours and leisure time.

Consider normal walking speed at which you can still talk **without being out of breath.**

3. USE OF FOOD SUPPLEMENTS AND SIMILAR PRODUCTS

IMPORTANT: Food supplements are products intended to supplement the normal diet. They are concentrated sources of nutrients or other substances with a physiological effect and can be found in the market in measured quantitative units. In practice, they can be capsules, lozenges, tablets and other similar forms (e.g. powders, bars, liquid in ampoules, in drop dispensing bottles and in other forms). This research also covers other similar products enriched with nutrients or other substances with a physiological effect, in the form of portion control packaging, e.g. protein bars, protein drinks, isotonic drinks, energy drinks, etc.

3.1 Did you consume any food supplements or similar products within the last year (12 months)?

3.1.1 Yes

3.1.2 No, I have not consumed any food supplements. You have completed the interview. Thank you for participating!

3.2 Where did you purchase the food supplements or similar products within the last year from? (Multiple possible answers)

3.2.1 Pharmacy

3.2.2 Online shop

3.2.3 Fitness centres and specialized shops

3.2.4 Food shops (such as Mercator, Spar, DM, Müller, also food shops abroad)

3.2.5 Post Exchange (PX) shops

3.2.6 Doorstep sales

3.2.7 Friends/acquaintances

3.2.8 Other (enter):

____

Please enter all the food supplements and similar products you have consumed within the last 12 months in the enclosed summary table on the following page. After that, continue with question **3.4**. You will answer a set of questions for each individual food supplement separately. It is important that you answer all the questions for each food supplement you consumed within the last year accurately.

When you have completed the set of questions for each individual food supplement, **turn the interview questionnaire to the last page** and answer the questions there.

Summary table on consumption of food supplements and similar products within the last year ID number:

No.	Product name and manufacturer (provide detailed description if the data is not available)	Form of food supplement	Your daily dose* (for example 2 capsules, 2 gels, etc.)	Photo**	Product website
1		3.3.1 capsule 3.3.2 powder 3.3.3 tablet<br>3.3.4 syrup 3.3.5 gel 3.3.6 bar<br>3.3.7 fizzy tablet 3.3.8 drops<br>3.3.9 spray 3.3.10 plastic bottle/can<br>3.3.11 other: ________________________		YES<br>NO	
2		3.3.1 capsule 3.3.2 powder 3.3.3 tablet<br>3.3.4 syrup 3.3.5 gel 3.3.6 bar<br>3.3.7 fizzy tablet 3.3.8 drops<br>3.3.9 spray 3.3.10 plastic bottle/can<br>3.3.11 other: ________________________		YES<br>NO	
3		3.3.1 capsule 3.3.2 powder 3.3.3 tablet<br>3.3.4 syrup 3.3.5 gel 3.3.6 bar<br>3.3.7 fizzy tablet 3.3.8 drops<br>3.3.9 spray 3.3.10 plastic bottle/can<br>3.3.11 other: ________________________		YES<br>NO	
4		3.3.1 capsule 3.3.2 powder 3.3.3 tablet<br>3.3.4 syrup 3.3.5 gel 3.3.6 bar<br>3.3.7 fizzy tablet 3.3.8 drops<br>3.3.9 spray 3.3.10 plastic bottle/can<br>3.3.11 other: ________________________		YES<br>NO	

* Please enter your daily dose on the day when you consumed the food supplement or similar product. ** Please mark YES if you have the product with you or if you have a photo of the product. ***Note: if there are more supplements consumed, add an identical row from the table for each

FOOD SUPPLEMENTS no. 1* (*sequence number from the summary table)

3.4 Please mark how often you consumed this food supplement within the last year, for <u>each season</u> separately.

	Several times a day	Once a day	4-6 times per week	1-3 times per week	1-3 times per month	Less frequently	Never
3.4.1 Winter							
3.4.2 Spring							
3.4.3 Summer							
3.4.4 Autumn							
	A	B	C	D	E	F	N

3.5 Please mark whether you consumed the mentioned food supplement in any of the periods featured below within the last year.

*Note: Please select 'not applicable' if you were not in such situation within the last year (for example, if you have not been on a military mission within the last 12 months, mark 'not applicable' when answering 'during a military mission').

	Yes	No	Not applicable*
3.5.1 During a period which required greater physical capability			
3.5.2 During a military operation abroad			
3.5.3 During movement skills assessment			
3.5.4 During an illness			
	A	B	N

3.6 Please identify the reasons for your decision to use this food supplement? (Multiple possible answers)

3.6.1 Deficiency of certain substances in my regular nutrition

3.6.2 Increased physical capability (strength, muscles, gaining muscle mass)

3.6.3 Recovery
3.6.4 Improving the functioning of the immune system
3.6.5 Increased concentration and/or mental capacity
3.6.6 Rehydration or electrolyte replenishment
3.6.7 Improved metabolism of nutrients
3.6.8 Reducing fatigue
3.6.9 Prevention of drowsiness
3.6.10 Improved sleep
3.6.11 Lack of appetite
3.6.12 Weight management
3.6.13 Bone and/or teeth health
3.6.14 Skin, hair and/or nail health
3.6.15 Other (enter): ______________________________

3.7 Who recommended you take an individual food supplement from the summary table?

	1	2	3	4	5	6	7	8	9	10
3.7.1 Consumption at my own discretion										
3.7.2 Doctor										
3.7.3 Pharmacist										
3.7.4 Other expert (for example a fitness coach, etc.)										
3.7.5 Co-workers										
3.7.6 Relatives, friends, acquaintances										
3.7.7 Commercials (TV, radio, etc.)										
3.7.8 Online information										
3.7.9 Other: ____________										
	1	2	3	4	5	6	7	8	9	10

3.8 Have you noticed any <u>positive</u> effects while consuming an individual food supplement from the summary table?

	**1**	**2**	**3**	**4**	**5**	**6**	**7**	**8**	**9**	**10**
3.8.1 Yes, the effects were noticeable.										
3.8.2 The effects were hardly perceptible.										
3.8.3 There were no positive effects.										
	1	2	3	4	5	6	7	8	9	10

3.9 Have you noticed any <u>negative</u> effects while consuming an individual food supplement from the summary table? (Please, mark the effects you have noticed) (Multiple possible answers)

	**1**	**2**	**3**	**4**	**5**	**6**	**7**	**8**	**9**	**10**
3.9.1 Headache										
3.9.2 Nausea										
3.9.3 Digestive disorders										
3.9.4 Skin problems										
3.9.5 Cardiovascular problems										
3.9.6 Allergic reactions										
3.9.7 Reduced general wellbeing										
3.9.8 Reduced mental wellbeing										
3.9.9 Other: _______________										
3.9.10 No negative effects										
	1	2	3	4	5	6	7	8	9	10

Thank you for participating!

Supplementary Table S1: Number and frequency of study participants who used supplements based on demographic and anthropometric data (n=448)

		Vitamin-mineral	p-value χ2 a	Vitamin supplements b	p-value χ2 a	Mineral supplements b	p-value χ2 a	Protein supplements b	p-value χ2 a	Fatty acids supplements b	p-value χ2 a	Energy drinks and caffein	p-value χ2 a	Sports drinks and similar	p-value χ2 a	Creatine b	p-value χ2 a	Herbal supplements b	p-value χ2 a
**All**		**46%**		**36%**		**30%**		**36%**		**18%**		**19%**		**9%**		**5%**		**4%**	
**Sex**	Male	45%	ns	35%	ns	29%	ns	37%	ns	18%	ns	19%	ns	10%	ns	6%	ns	3%	ns
	Female	53%		45%		37%		31%		16%		12%		8%		0%		12%	
**Military mission**	Yes	36% *a*	<0.0001	31% *a*	0.0118	21% *a*	0.0001	29% *a*	0.0019	15%	ns	25% *b*	0.0002	7%	ns	4%	ns	3%	ns
	No	55% *b*		42% *b*		38% *b*		43% *b*		21%		11% *a*		11%		6%		5%	
**Age**	18-29 years	39% *a*	0.0059	31% *ab*	0.0146	27% *ab*	0.0235	35%	ns	16%	ns	29% *b*	0.0005	7%	ns	11%	na	5%	na
	30-39 years	54% *b*		44% b		36% *b*		32%		22%		21% *ab*		10%		6%		5%	
	≥ 40 years	39% *a*		31% *a*		23% *a*		41%		14%		10% *a*		10%		1%		2%	
**Rank**	Private	44%	ns	34%	ns	31%	ns	32%	ns	19%	ns	21%	ns	7%	ns	7%	na	4%	na
	Non-commissioned officer	43%		37%		25%		43%		18%		13%		9%		4%		5%	
	Higher ranks	54%		45%		33%		37%		16%		21%		16%		1%		1%	
**Education**	Lower secondary school	30% *a*	0.013	20% *a*	0.011	25%	ns	39%	ns	11%	ns	25%	ns	5%	na	2%	na	2%	na
	Secondary school	44% *b*		35% *ab*		28%		37%		20%		17%		8%		6%		4%	
	Higher education	55% *b*		45% *b*		35%		33%		17%		20%		15%		3%		3%	
**BMI**	<25	50%	ns	39%	ns	35%	ns	36%	ns	16%	ns	17%	ns	10%	ns	7%	na	9%	na
	25-30	43%		35%		28%		35%		18%		19%		10%		5%		2%	
	>30	48%		38%		25%		41%		25%		20%		5%		2%		2%	
**MSAM score**	<3	39%	ns	34%*ab**	0.0495	22%	ns	39%	ns	15%*ab**	0.0312	11%	ns	5%	ns	1%	na	2%	na
	4	40%		27% *a**		29%		32%		11% *a**		18%		6%		5%		2%	
	5	50%		41% *b**		33%		37%		22% *b**		21%		12%		6%		5%	
**Physical activity**[1]	Low	35%	ns	35%	ns	24%	ns	54%	ns	20%	ns	37%a	<0.0001	6%	na	4%	na	7%	na
	Medium	46%		38%		30%		34%		18%		17%b		10%		6%		31%	
	High	51%		36%		34%		39%		18%		8%c		12%		2%		5%	

na – not assesed; ns – no statistically significant differences; a – Pearson's χ2 test with continuity correction; b – Proportions data comparisons using sample test for equality of proportions with continuity correction: p-values adjusted with Holm procedure; (*) p-values not adjusted

[1]Self reported physical activity, assigned using DEEPA (Daily Energy Expenditure for Physical Activity) score

Supplementary Table S2: Self-reported reason for using food supplements and similar products (FSSP) for study participants recruited from the Slovenian Armed Forces

	Vitamin/ mineral suppleme nts	Vitamin suppleme nts	Mineral suppleme nts	Protein suppleme nts	Faty acids suppleme nts	Energy drinks, caffeine suppleme nts	Sports drinks and similar
Deficiency of certain substances in my regular nutrition	60%	44%	69%	49%	83%	5%	15%
Increased physical capability	19%	14%	31%	68%	26%	16%	34%
Recovery	47%	36%	75%	79%	33%	6%	51%
Improving the functioning of the immune system	66%	40%	44%	29%	52%	6%	13%
Increased concentration and/or mental capacity	15%	3%	-	4%	13%	23%	9%
Rehydration or electrolyte	23%	43%	31%	16%	5%	16%	66%
Improved metabolism of nutrients	13%	5%	13%	9%	21%	3%	4%
Reducing fatigue	23%	13%	13%	20%	9%	60%	17%
Prevention of drowsiness	2%	1%	-	1%	-	61%	4%
Improved sleep	8%	1%	13%	2%	-	-	-
Lack of appetite	-	-	-	1%	-	-	-
Weight management	11%	2%	13%	26%	20%	8%	2%
Bone and/or teeth health	11%	8%	25%	3%	10%	-	-
Skin, hair and/or nail health	11%	7%	6%	1%	12%	-	-

Supplementary Table S3. Prevalence of use of food supplements and similar products (FSSP) and odds ratios (95% confidence interval) for study participants recruited from the Slovenian Armed (12-month period)

**Constituent**	**N**	**Prevalence**	**Prevalence (among users of FSSP)**	**Median**	**Mean**	**SD**
Omega-3 fatty acids	79	18%	26%	0.61 g	1.19	1.39
- EPA/DHA	70	16%	23%	N/A		
Proteins and amino acids	205	46%	67%	N/A		
L-carnitine	15	3%	5%	N/A		
Creatine	39	9%	13%	N/A		
Vitamins	243	54%	80%	N/A		
- Vitamin B6	202	45%	18%	4 mg	10.7	59.0
- Vitamin B12	190	42%	23%	5 µg	35	147
- Vitamin C	180	40%	57%	80.5 mg	270	483
- Niacin	178	40%	10%	16.3 mg	29.7	42.6
- Vitamin E	174	39%	59%	10.4 mg	27.8	96.0
- Riboflavin	168	38%	54%	1.4 mg	6.7	30.2
- Thiamin	164	37%	55%	1.1 mg	6.6	34.8
- Pantothenic acid	150	33%	59%	6.00 mg	13.2	35.4
- Folic acid	145	32%	66%	200 mg	282	279
- Biotin	73	16%	49%	50 µg	145	239
- Vitamin D	69	15%	24%	12.5 µg	21.5	29.8
- Vitamin A	55	12%	48%	1050 µg	1666	2026
- Vitamin K	29	6%	63%	75 µg	108	102
Minerals	222	50%	73%	N/A		
- Mg	186	42%	30%	200 mg	293	402
- Ca	129	29%	42%	160 mg	329	824
- K	92	21%	61%	130 mg	260	426
- Zn	69	15%	14%	15 mg	21.4	26.0
- Fe	58	13%	19%	7.0 mg	13.2	14.3
- Se	55	12%	11%	55 µg	69	56
- Cr	50	11%	23%	55.0 µg	165	294
- Mn	45	10%	14%	2 mg	3.2	3.9
- Cu	44	10%	15%	1 mg	1.5	1.9
- P	42	9%	16%	141 mg	360	934
- I	32	7%	9%	150 µg	173	147
- Mo	27	6%	18%	30 µg	57	91
Caffeine	94	21%	31%	170 mg	198	282
Flavonoids and polyphenols	15	3%	5%	N/A		
Coenzyme Q10	10	2%	3%	N/A		
Lycopene	19	4%	6%	N/A		
Lutein/Zeaxanthin/Astaxanthin	17	4%	6%	N/A		
Botanical ingredients	69	15%	23%	N/A		

Notes: N/A – not assessed
